# Ca^2+^ addition facilitates the shell repair with eggs production of *Pomacea canaliculata* through biomineralization and food intaking regulation

**DOI:** 10.1038/s41598-023-43071-4

**Published:** 2023-10-23

**Authors:** Yingtong Chen, Fucheng Yao, Jiaen Zhang, Chunxia Zhang, Zhong Qin, Jing Guo

**Affiliations:** 1 Guangdong Provincial Key Laboratory of Eco-Circular Agriculture, South China Agricultural University, Guangzhou, 510642 China; 2 Department of Ecology, College of Natural Resources and Environment, South China Agricultural University, Guangzhou, 510642 China; 3 Guangdong Engineering Research Center for Modern Eco-Agriculture and Circular Agriculture, Guangzhou, 510642 China; 4 Key Laboratory of Agro-Environment in the Tropics, Ministry of Agriculture and Rural Affairs, Guangzhou, 510642 China; 5 Henry Fok School of Biology and Agriculture, Shaoguan University, Shaoguan, 512005 China

**Keywords:** Ecology, Biodiversity

## Abstract

*Pomacea canaliculata* was by far one of the most harmful invasive organisms in the world, causing serious harm to aquatic crops and ecosystem. Calcium carbonate is a common component of aquatic environment, which is important for the growth of *Pomacea canaliculata*. Therefore, the objective of this study was to investigate the response characteristics of *P. canaliculata* suffered shell breakage to the addition of calcium carbonate in water environment. In this experiment, we explored the effects of calcium carbonate addition on the *P. canaliculata* shell repair rate, food intake, egg production, shell strength, and calcium content through breaking the snails shell and the addition of calcium carbonate treatment. The results showed that snail broken-shell repaired mostly within 21 days. The snails experienced a significant increase in shell repair rates during earlier days of the treatment, especially for female snails. Food intake of snails exhibited different patterns when their shells were broken and calcium carbonate was added. Shell breakage treatment combined with calcium carbonate addition significantly increased the diameter of snail eggs compared with the control and the calcium carbonate addition treatment without shell-broken snail group. There was no significant difference in shell strength or calcium content of male snails between the treatments. The study suggests that *P. canaliculata* exhibits a sex-dependent response pattern when subjected to shell damage and calcium carbonate addition. The findings can provide some references to better understand the invasion mechanism and survival strategy of the *P. canaliculata*.

## Introduction

*Pomacea canaliculata* is a globally invasive snail species. This species was introduced to China from Argentina in the 1980s for economic purposes as a food source. However, these snails were subsequently abandoned in natural aquatic environments and rice fields due to their unpalatability and their role as carriers of the pathogenic parasite *Angiostrongylus cantonensis*. This situation resulted in significant agricultural losses in southern China, particularly in rice production^1^. The remarkable adaptability of *P. canaliculata* populations to their surroundings enables them to proliferate within aquatic ecosystems, posing a substantial threat to local biodiversity^2^.

Currently, snail control primarily relies on chemical methods, which unfortunately result in significant ecological harm and the snail's pesticide resistance due to prolonged use of chemicals. In recent years, notable progress has been made in the biological management of *P. canaliculata*, taking advantage of its natural predators, a strategy well-suited for China with its prevalent biodiversity. The majority of these predators target the snails by breaking their shells and consuming the soft tissues within the shells. Some researchers observed that the size of the mouth in *Trachemys scripta elegans* correlates directly with the size of the snails they prey upon. Certain predators are restricted to prey on smaller snails due to limitations in their biting power and size^3,4^. Consequently, the strength of the snail's shell emerges as a pivotal factor influencing the success of predation by its natural enemies.

The shell is the result of biomineralization, composed primarily of minerals and organic molecules, with calcium carbonate constituting 90% of its composition^5,6^. Biomineralization is a meticulously orchestrated process in which the mantle assumes a crucial role^7^. The mantle of mollusks promotes calcium accumulation during shell formation, overseeing neurosecretion to regulate shell growth by secreting an organic matrix that aids in calcium carbonate deposition, a pivotal factor in shell formation^8,9^. Microscopic examinations by researchers revealed that the molluscan mantle undergoes a series of metamorphic stages. Notably, the juvenile phase emerges as a critical period for mantle development in mollusk growth. Furthermore, observations showed enhanced mitochondrial and endoplasmic reticulum development in the epithelial cells at the mantle's periphery, with the central region characterized by increased cytoplasm and fewer organelles. This suggests that the reparative capacity of the mantle's edge surpasses that of the central region, with repair rates potentially influenced by environmental conditions^10,11^.

Shell repair rate serves as a vital metric for evaluating the snail's proficiency in shell restoration^12^. Measuring the shell repair rate allows us to discern the speed and effectiveness of shell restoration within various treatment groups^13^. Feeding rate, quantifying the snails' food consumption, serves as an indicator of their activity and growth status^14,15^. Comparative analysis of feeding rates among distinct treatment groups facilitates an examination of the feeding patterns and food utilization capabilities of *P. canaliculata* subjected to shell breakage stress.

Egg diameter serves as a key metric for evaluating the reproductive capacity of *P. canaliculata*^16,17^. Measuring egg diameter provides insights into the reproductive strategies and fertility of snails subjected to shell breakage stress in various treatment groups^18^. Shell strength and calcium content serve as indicators for assessing shell quality and calcium metabolism in snails^19^. Comparative analysis of shell strength and calcium content among distinct treatment groups enables us to understand changes in shell quality and calcium metabolism in snails exposed to shell breakage stress^20,21^.

Environmental factors exert a profound influence on mollusks' biomineralization process, especially concerning shell repair. Biomineralization is a meticulously regulated process, characterized by intricate interactions among diverse internal factors^22^. Calcium ions play a pivotal role in biomineralization and hold particular significance in mollusk shell repair. Environmental calcium ion concentrations directly influence the accessibility of calcium ions to mollusks, its elevation enhances the mollusks' capacity to absorb and utilize calcium ions, thereby expediting shell repair and formation^23^.

Temperature, pH, nutrient availability, salinity, environmental pollution, and oxidative stress are factors that exert influence on the biomineralization process^24–29^. The cumulative impact of environmental factors on mollusks' biomineralization process is intricate and substantial. Favorable environmental conditions can facilitate the seamless advancement of biomineralization, whereas adverse conditions may hinder shell repair and growth, consequently impacting mollusks' survival and reproductive abilities^30–36^.

In this study, we devised four distinct experimental treatments to explore the adaptive strategies of *P*. *canaliculata* when subjected to shell breakage stress. We conducted tests on the shell repair rate, food intake, egg diameter, shell strength, and calcium content of the snails. Calcium carbonate, a pivotal contributor to the shell formation process of *P. canaliculata*, was intentionally selected as a variable. Thus, by manipulating the addition of calcium carbonate and the snail shell breakage treatment, we aimed to gain a more comprehensive understanding of snail adaptation and response to shell repair under stressful conditions. Furthermore, monitoring changes in calcium content in *P. canaliculata* can elucidate the regulatory mechanisms of biochemical substances necessary for the shell repair process. Examining the relationships between these indicators can assist us in unveiling the ecological adaptability of *P. canaliculata* in response to shell breakage stress and in substantiating the presence of their resilient survival mechanisms and regulatory processes.

## Materials and methods

### Experimental materials

We gathered all tested *P. canaliculata* individuals from irrigation ditches on a farm situated at South China Agricultural University in Guangzhou, Guangdong Province, China (23° 14′ N, 113° 38′ E). The snails were then reared in plastic aquaria measuring 45 cm × 35 cm × 35 cm, submerged to water with an approximate depth of 20 cm. They were provided with daily feedings of fresh lettuce within a climate-controlled indoor balcony.

### Experimental Performance

Following several days of rearing, we randomly selected healthy adult *P. canaliculata* of comparable size (shell height 25 ± 5 mm, weight 5.3 ± 0.5 g). After differentiating males and females, we marked their shells using a waterproof pen. We utilized twelve plastic aquariums, each filled with 15 cm of water and containing 10 male and 10 female snails. We employed analytical grade (AR) calcite powder as the source of calcium carbonate, adding 15 g per day. The treatments comprised a shell-broken snail group with calcium carbonate addition, a shell-broken snail group without calcium carbonate addition, a calcium carbonate addition treatment without shell-broken snail group, and a blank control snail group without shell-broken treatment and calcium carbonate addition. We installed screens on the water surface to prevent the escape of *P. canaliculata* and promptly removed any deceased snails. We provided an ample daily supply of lettuce as the primary food source for the snails. We replaced water in plastic aquariums using tap water weekly, retaining approximately 3/4 of the original volume to maintain water clarity. We measured the water temperature daily at 16:00 using a thermostat positioned approximately 5 cm below the water surface with an accuracy of 0.1 °C. Eggs produced on the same day were promptly collected. The experiment spanned 21 days, during which the snails were fed once daily at 16:00, and any remaining lettuce was removed before the subsequent feeding. The experimental treatments and groupings are shown in Table 1.

**Table 1:** Experimental grouping and treatment.

Number	Group	Treatment
1	SC	A shell-broken snail group with calcium carbonate addition
2	S	A shell-broken snail group without calcium carbonate addition
3	C	Calcium carbonate addition treatment without shell-broken snail group
4	CK	Blank control snail group without shell-broken treatment and calcium carbonate addition

### Shell strength measurement

Dissecting scissors and a scalpel were employed to delicately separate the snail's shell from its flesh without causing damage. We utilized an Edbaura pressure testing machine equipped with a digital display push–pull meter, possessing an accuracy of 0.1 N, to ascertain the shell's compressive capacity. For testing, the shell was positioned flat on the platform with the operculum facing downward. Subsequently, the machine applied pressure to the shell until it fractured, at which point the operation was terminated. The numerical value displayed at this juncture represented the shell's strength.

### Shell repair observation and calculation

Photographs of each shell-broken snail were taken at the time points of 0, 2nd, 4th, 6th, 8th, 11th, and 21st day. Subsequently, the width of the clipped shell and height of the shell repair was quantified using Vernier calipers, after which the snails were returned to their respective aquariums. The shell repair area and repair rate are calculated according to the following equations:

$$ \begin{aligned}  & {\text{Area of repair (AR)}} = {\text{W}} \times {\text{H}} \hfill \\ & {\text{Repair rate }}\left( {{{\%}}} \right) = {1}00\% \times \left( {{\text{ARl}} - {\text{ARf}}} \right)/{\text{Act;}} \hfill \\ \end{aligned}$$

“W” indicates the width of the clipped shell; “H” indicates the height of the shell repair; “ARl” indicates the area of shell repair for the latter measurement; “ARf” indicates the shell repair area of the former measurement; “Act” denotes the area of the shell cutted off at the beginning of the test.

### Measurement of snail eggs

We collected fresh egg masses daily, recorded their weight and quantity, and individually stored each egg mass in a dedicated container. After the experiment concluded, we immersed the egg masses in a 2% NaOH solution for 1.5 h. We measured the diameter (in millimeters) of each egg by randomly selecting 10 eggs and assessing their dimensions with a Vernier caliper (Model: DL91150).

### Measurement of food intake

Before feeding, excess water on the surface of lettuce was removed. Feeding amounts were recorded on days 2, 3, 8, 12, and 19 following the experimental treatment. Fresh lettuce, pre-weighed, was provided to each experimental group. After 24 h, the remain lettuce was collected, drained, and weighed again to calculate the snails' food intake. The food intake for each group was determined in relation to the survival count.

$$\begin{gathered} {\text{Total food intake}}\left( {\text{g}} \right) = {\text{Total weight of the lettuce fed}}\left( {\text{g}} \right){-}{\text{Total weight of the remain lettuce next day}}\left( {\text{g}} \right); \hfill \\ {\text{Food intake}}\left( {\text{g}} \right) = {\text{Total food intake}}\left( {\text{g}} \right)/{\text{Number of snail survivors on the day}}. \hfill \\ \end{gathered}$$

### Measurement of calcium and protein content

We assessed calcium content and protein concentration using kits from Nanjing Jiancheng Bioengineering Institute. Calcium content was determined using the methyl thymol blue method, while total protein concentrations (TP) were determined via the Bicinchoninic Acid (BCA) method^37,38^. The procedures were conducted as follows: (1) preparation of the test solution: accurately weighed mantle tissue (100 mg) was mixed with physiological saline at a ratio of weight (g):volume (mL) = 1:9. The mixture was homogenized under ice-water bath conditions at 2500 rpm and centrifuged for 10 min. The resulting supernatant was retained for testing or further dilution. (2) Calcium and protein content determination: followed the kit's specified procedures.

$$\mathrm{The \,calcium \,content}\,(\mathrm{mmol}/\mathrm{gprot})=\frac{A\left(Treatment\,\right)-A\left(Blank\right)}{A\,\left(Standard\right)-A\left(Blank\right)}\times \mathrm{C}(Standard)/TP$$

$$\mathrm{TP}\,(\mathrm{g}/\mathrm{L})=\frac{A\left(Treatment\right)-A\left(Blank\right)}{A\left(Standard\right)-A\left(Blank\right)}\times \mathrm{C}\left(Standard\right)\times N$$

A(Treatment): tested data for each treatment group. A(Blank): tested data for blank group. C(Standard): standard concentration, 1 mmol/L.  TP: total protein content. N: pre-test dilution times of a sample.

### Statistical analysis

Statistical analyses were conducted using SPSS version 20.0. Normality analysis was performed on male and female snails within the same treatment and at the same time. To assess the impact of calcium carbonate and shell breakage on shell repair in *P. canaliculata*, we employed repeated-measures ANOVA and Duncan's multiple comparisons to analyze the repair rates, food intake, shell strength and calcium content across various treatments. Additionally, we analyzed the differences in shell repair rates, shell strength and calcium content between male and female snails under the same treatments at the end of the experiment using t-tests. Graphs were created using Origin 8.0. Significance was set at *P* < 0.05, or at *P* < 0.01.

## Result

### Shell repair rates of *P. canaliculata* in different treatments

**Figure 1:**
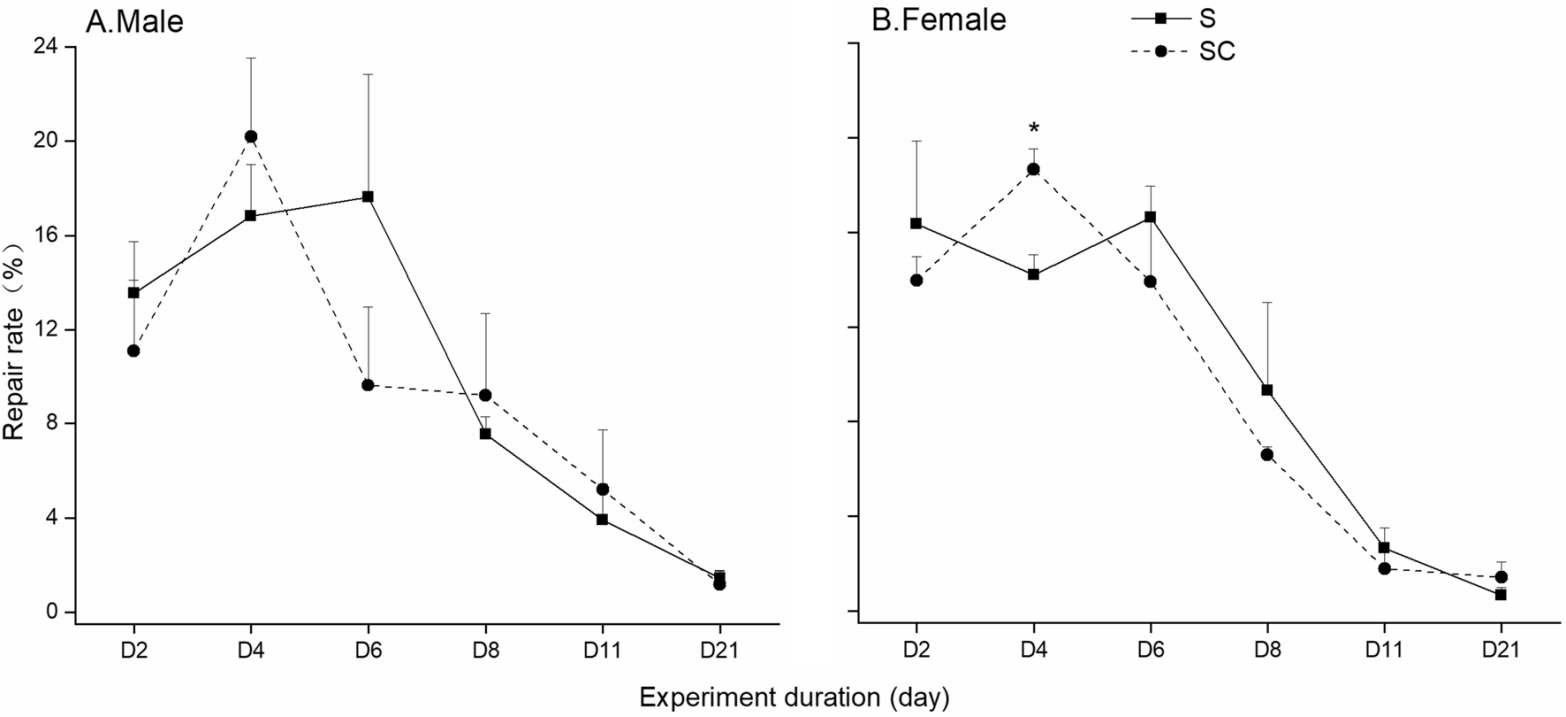
Shell repair rate of *P. canaliculata* in different treatments with different genders. SC and S refer to shell-broken snail group with calcium carbonate addition, and shell-broken snail group without  calcium carbonate addition, C and CK refer to calcium carbonate addition treatment without shell-broken snail group and blank control snail group without shell-broken treatment and calcium carbonate addition, respectively. *Indicates significant differences between treatments at the same time.

The snail shell repair rate exhibited an initial increase followed by a gradual decline, reaching its lowest point at day 21 under both treatments (Fig. 1). Notably, only in the fourth day, we observed significant differences in female snails between the two treatments, with the SC snail group showing significantly higher values than the S group (*P* < 0.05). In the S group, both males and females displayed an average repair area of 1.0 ± 0.02 mm^2^ per day. On the day 2, this rate was 14.2% for males and 16.8% for females, followed by an upward trend in the early period, peaking at 17.6% for males and 16.6% for females on day 6. Subsequently, a significant decrease occurred until the end of the experiment (*P* < 0.05), with rates on the final day at 1.4% for males and merely 0.6% for females. In the meantime, the SC group displayed a pattern of increase followed by decrease in both males and females, with identical average repair area of 0.9 ± 0.02 mm^2^ per day. In this group, the rate significantly increased (*P* < 0.05) from the 2nd to the 4th day, with 9.6% for males and 13.9% for females on the second day, and peaking at 20.9% and 18.6% on the fourth day, respectively. The repair rates exhibited a sharp decrease on the final day, at 1.2% for males and 1.4% for females (*P* < 0.01). Repeated-measures ANOVA results indicated no significant effect of calcium carbonate addition on the rate of shell repair (*F*_1,8_ = 0.018, *P* > 0.05).

### Food intake of *P. canaliculata* in different treatments

**Figure 2:**
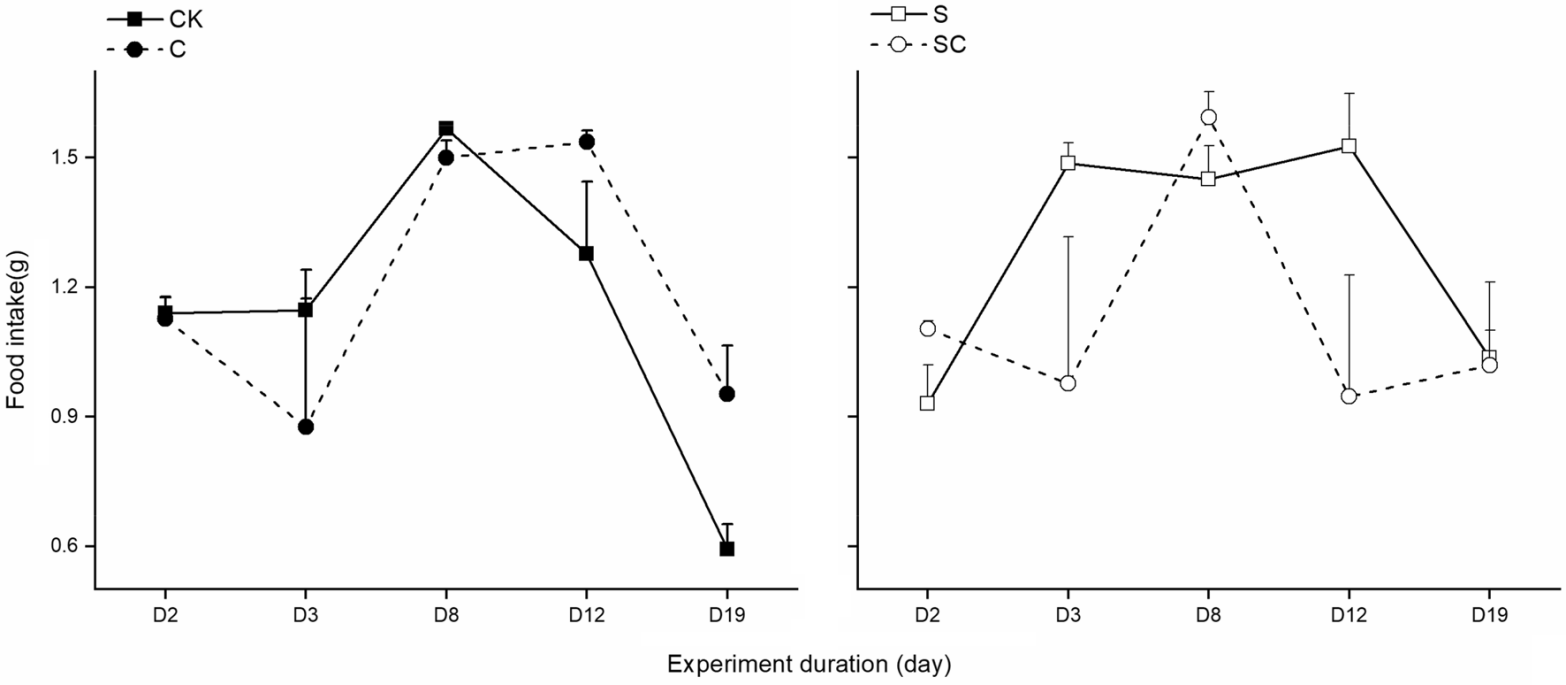
Food intake of *P. canaliculata* under different treatments. SC and S refer to shell-broken snail group with calcium carbonate addition, and shell-broken snail group without calcium carbonate addition, C and CK refer to calcium carbonate addition treatment without shell-broken snail group and blank control snail group without shell-broken treatment and calcium carbonate addition.

The food intake of the *P. canaliculata* substantially exhibited a "first increasing, then decreasing" trend over time in C and CK groups (Fig. 2). In the CK group, the feeding rate significantly increased on day 8 but decreased from day 8 to the end of the experiment. Similarly, the C group displayed a significant increase in food intake from the day 3 to  day 12, followed by a subsequent decrease. In S group, food intake showed a change trend of "first increasing, then keeping stable, finally decreasing" with the highest cumulative feeding amount. In the SC group, food intake kept a relative stable level, while it reached a peak with amount of 1.59 g on day 8. Notably, there were no significant differences in food intake between the CK and C groups during the first two days of the experiment. Repeated-measures ANOVA indicated a significant impact of the interaction between calcium carbonate addition and shell breakage on the food intake of snails (*F*_1,8_ = 7.453, *P* < 0.05).

### Egg production of the *P. canaliculata* in different treatments

**Figure 3:**
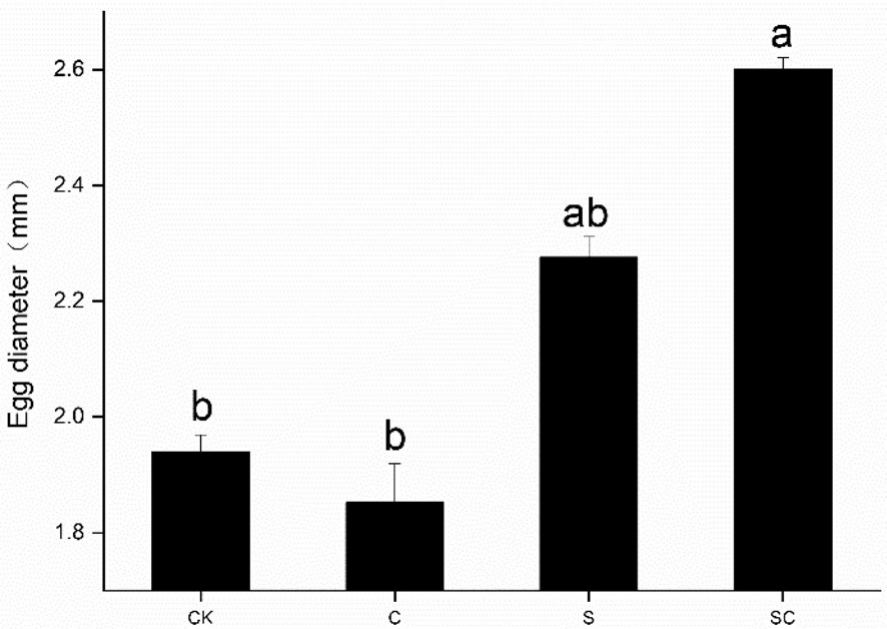
Egg diameter of *P. canaliculata* under different treatments. SC and S refer to shell-broken snail group with calcium carbonate addition, and shell-broken snail group without calcium carbonate addition, C and CK refer to calcium carbonate addition treatment without shell-broken snail group and blank control snail group without shell-broken treatment and calcium carbonate addition. Different lowercase letters in the graph indicate significant differences between different treatments.

Throughout the experiment period, the CK snail group produced 2 egg masses, each containing an average of 25.0 ± 4.0 eggs; the C group also produced 2 egg masses, with an average of 79.5 ± 40.5 eggs per mass; the S group yielded 4 egg masses, averaging 62.5 ± 12.6 eggs per mass, while the SC group produced 6 egg masses, each containing an average of 65.83 ± 24.4 eggs. The average egg diameters were 1.94 ± 0.03 mm, 1.85 ± 0.06 mm, 2.23 ± 0.04 mm, and 2.60 ± 0.02 mm in the CK group, C group, S group, and SC group, respectively (Fig. 3). Notably, the egg diameter in the SC group was significantly larger than that in the CK and C groups. Repeated-measures ANOVA revealed a highly significant change in egg diameter due to shell breakage (*F*_1,16_ = 164.574, *P* < 0.001), indicating that the snails with damaged shells produced larger eggs in diameter than those with unbroken shells. Additionally, the input of calcium carbonate led to a significant difference in egg diameter (*F*_1,16_ = 7.801, *P* < 0.05).

### Shell strength of *P. canaliculata* in different treatments

**Figure 4:**
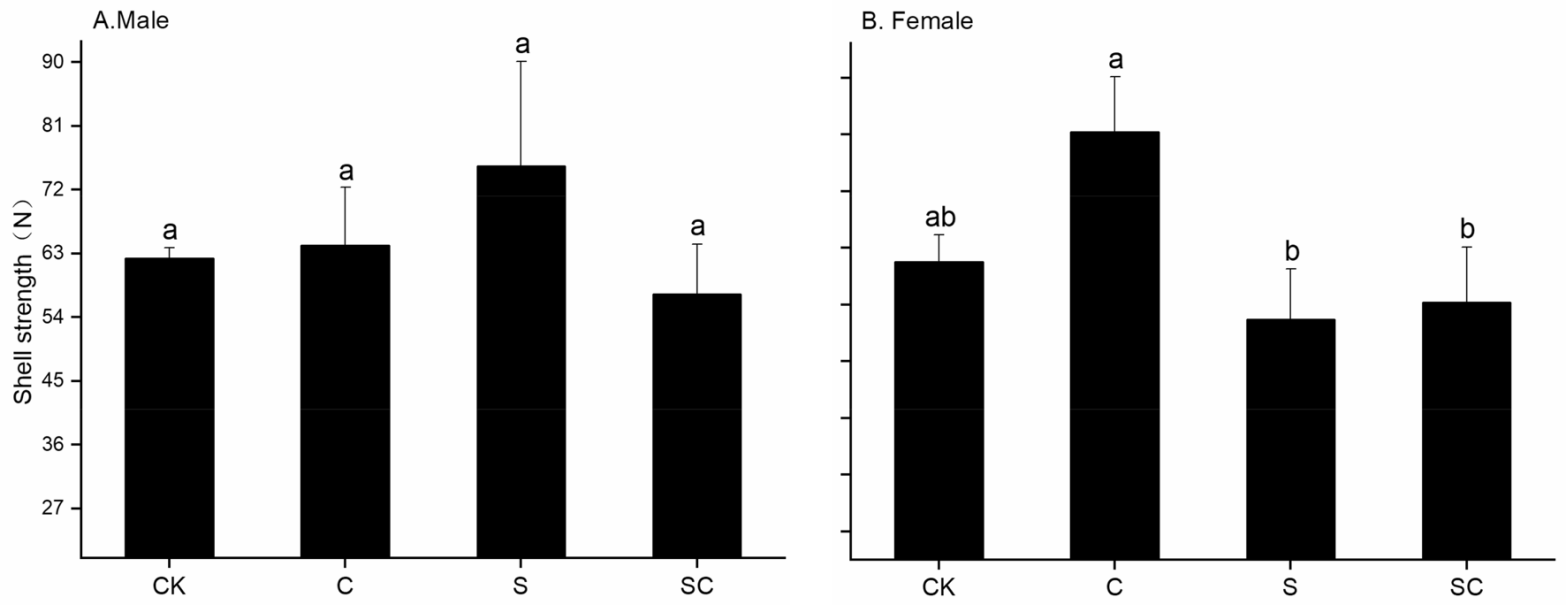
Shell strength of different sexes of *P. canaliculata* under different treatments. SC and S refer to shell-broken snail group with calcium carbonate addition, and shell-broken snail group without calcium carbonate addition, C and CK refer to calcium carbonate addition treatment without shell-broken snail group and blank control snail group without shell-broken treatment and calcium carbonate addition. Different lowercase letters in the graphs indicate significant differences under the same sex between different treatments.

A partially significant variation in snail shell strength was observed among the female groups (Fig. 4). The range of shell strength for male snails across all four treatments was from 57 to 75 N. Female snails, on the other hand, exhibited the highest shell strength of 80 N in the C group, which was significantly greater than the S group (53 N) and the SC group (56 N). Interestingly, there were no significant differences in shell strength between male and female snails within the same treatment. Repeated-measures ANOVA indicated the absence of significant differences in shell strength related to calcium carbonate addition (*F*_1,40_ = 0.081, *P* > 0.05), shell breakage treatment (*F*_1,40_ = 1.286, *P* > 0.05), and gender (*F*_1,40_ = 0.037, *P* > 0.05).

### Calcium content of *P. canaliculata* in different treatments

**Figure 5:**
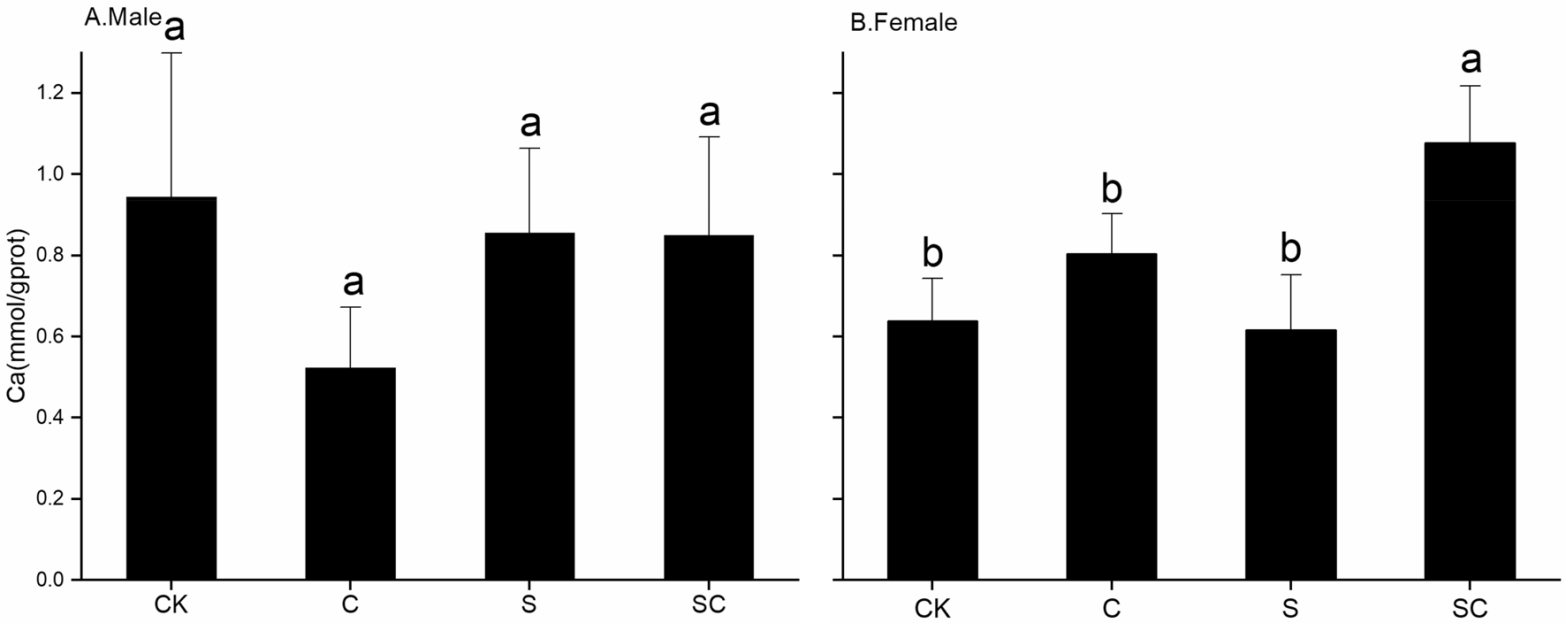
Calcium content of *P. canaliculata* under different treatments. Different lowercase letters indicate significant differences under the same sex between different treatments. SC and S refer to shell-broken snail group with calcium carbonate addition, and shell-broken snail group without calcium carbonate addition, C and CK refer to calcium carbonate addition treatment without shell-broken snail group and blank control  snail group without shell-broken treatment and calcium carbonate addition.

The total protein concentration of snails under all the treatments was determined using the BCA method (Table 2). Calcium content in the mantle under each treatment is presented in Fig. 5. In the CK group, male snails had a mantle calcium content of 0.94 ± 0.35 mmol/g prot, while female snails had 0.64 ± 0.10 mmol/g prot. Male snails in the C group exhibited the lowest calcium content at 0.52 ± 0.15 mmol/g prot. Notably, female snails in the SC treatment had significantly higher mantle calcium content than those in the other three treatment groups after 21 days of treatment. Repeated-measures ANOVA indicated no significant differences in calcium content caused by gender (*F*_1,40_ = 0.004, *P* > 0.05), shell breakage (*F*_1,40_ = 0.776, *P* > 0.05), or calcium carbonate addition (*F*_1,40_ = 0.132, *P* > 0.05).

**Table 2:** Total protein content (TP) of *P. canaliculata* mantle under different treatments (g/L).

Sex	Sample size	CK	C	S	SC
Male	6	2.81 ± 0.54	8.73 ± 5.54	2.33 ± 0.58	2.62 ± 0.53
Female	6	2.70 ± 0.46	1.85 ± 0.21	6.83 ± 4.66	1.85 ± 0.29

## Discussion

**Figure 6:**
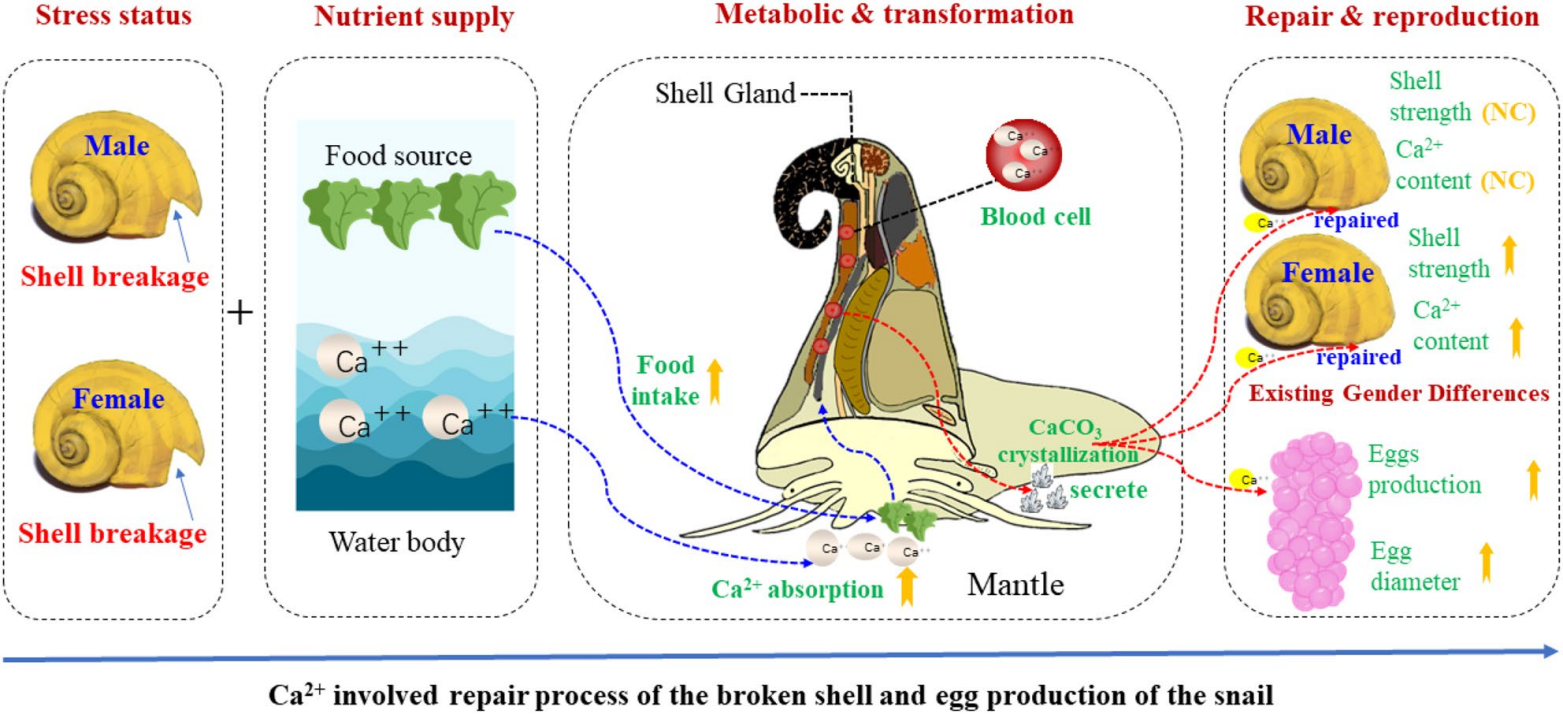
Ca^2+^ involved repair process of the broken shell and egg production of *P. canaliculata*. The blue dashed curved lines indicate the input process of materials. The red dashed curved lines indicate the output process of materials. The up arrows near the parameters mean the increase of values. The “NC” near the parameters means no change.

As depicted in Fig. 6, the broken-shell repair process and egg production of *P. canaliculata* are affected and regulated by several important factors, such as food and calcium supplies and other environmental conditions. In our study, the addition of calcium carbonate initially resulted in an increase in the snail shell repair rate, followed by a subsequent decrease. This trend can be attributed to the initial boost in calcium availability, facilitating the shell repair process. However, the repair process may reach a saturation point over time, limiting further improvement in the repair rate^39^. Furthermore, excessive calcium carbonate supplementation could adversely affect the snails by disrupting their acid–base balance or inducing toxic effects, ultimately reducing the shell repair rate^40^.

In the present study, we observed that shell-broken snails without calcium carbonate addition treatment had a higher level of food intake than the those with calcium carbonate addition treatment. The variations in the food intake of snails subjected to both shell damage and calcium carbonate addition may be attributed to several reasons. Firstly, it is likely that the shell damage induced stress responses in the snails, changing their usual feeding behavior^41^. In order to repair the broken shell, the snails needed to eat more lettuce to  supply calcium and offset the calcium deficiency when  no additional calcium was input. Meanwhile, the shell-broken female snails had higher calcium content in the calcium carbonate addition treatment than that in the treatment without calcium carbonate addition, further verifying that the injured snails can directly intake  calcium in the water, which may subsequently reduce their feeding amount. Secondly, the addition of calcium carbonate could lead to alterations in water chemistry, including an increase in pH, which may also affect snails feeding^42^.

In the snail group with both shell breakage and calcium carbonate addition treatment, a significant increase in egg diameter was observed. The combination of shell breakage and calcium addition can influence the physiological processes of *P. canaliculata*, impacting egg development and growth. Shell breakage likely initiates a cascade of responses within the organism, including the activation of cell proliferation and differentiation, particularly during egg development. Calcium, being a pivotal component of snail shells, plays a crucial role; with a sufficient calcium supply, snails can utilize calcium more effectively for egg shell development and reinforcement, resulting in larger egg diameters^43–46^. The augmentation in egg size may serve as an adaptive response, making it more challenging for predators to prey on the larger eggs, as observed in other species like *Diatraea saccharalis*, *Pomacentridae*, and *Trematomus bernacchii*^47–49^. Nonetheless, further investigations are required to unveil the related mechanisms governing this phenomenon. When exposed to the stress of shell damage, *P. canaliculata* likely undergoes a series of physiological and biochemical responses, including the activation of specific biochemical pathways to cope with external pressures^50–52^. These responses can lead to alterations in body fluid composition, subsequently affecting egg development and size. Additionally, the input of calcium carbonate serves as an additional source of calcium, facilitating the process of egg calcification^19^. Tahara *et al.* conducted experiments comparing body fluid composition and egg size across different environments^53^. Further research is essential to comprehensively investigate changes in the body fluid composition of injured *P. canaliculata* and to identify the signaling molecules involved in regulating egg size.

Among female snails, shell strength exhibited significant differences among the most treatments. The snail group without broken shells but treated with calcium carbonate addition had significantly highest shell strength, the reason would be that the supplementation of calcium carbonate contributes to increase calcium content and shell density of snails, thereby fortifying shell strength^54^. The control group, having undergone no specific treatment, demonstrated intermediate shell strength. Shell damage treatments likely influenced the integrity and structure of snail shells, resulting in reduced shell strength,  but the shell-broken snail group with calcium carbonate addition showed an increasing trend in shell strength, relative to the shell-broken snail group without  calcium carbonate addition, which may also indicate the contribution of calcium carbonate addition. Conversely, there were no significant differences in shell strength and calcium content among male snails across the various treatments. This variance between female and male snails might be attributed to the pronounced influence of hormonal factors and reproductive activities on snail shell strength and calcium content.

The shell repair capacity of mollusks has a direct bearing on their susceptibility to pests and diseases. In laboratory rearing conditions, all individuals of *Buccinum undatum* completely restored their damaged shells within 30 days^55^. *Pinctada fucata* exhibited significant shell repair within 5–20 days following shell edge damage^56^. The mantle, vital for mollusk biomineralization, is comprised of epithelial cells capable of secreting essential inorganic ions such as Ca^2+^ and CO_3_^2−^. It plays a pivotal role in snail shell regeneration^57^. The mantle is supposed to promptly allocate resources and energy for repairing damaged shells and tissues when breakage occurs. Snails initiate the growth of new shells as early as 1 day after shell breakage, often completely covering the damaged area within 5–10 days. The repair rate is notably high in the early stages but gradually diminishes as shell repair progresses. Notably, during the experiment, the addition of calcium to the water environment led to a more pronounced increase in the begining of shell repair process, likely due to the facilitation of the biomineralization process through calcium intake by snails, thereby improving the repair rate, especially for female snails. 

After suffered shell injuries, gastropods usually better allocate energy and safeguard themselves from environmental threats to minimize further harm and potential mortality when their delicate soft tissues become exposed^58^. The repair process is intricately governed by biomolecules, and in the case of oysters, shell matrix proteins (SMPs) play a pivotal role in regulating CaCO_3_ crystallization in vitro. However, the composition of the newly repaired shell differs from that of the original one^17^. Mollusks invest substantial energy and resources in shell repair, affecting their overall metabolic functioning during the post-injury period^59^. Spawning represents a critical stage in snail life history, with snails strategically allocating energy based on environmental nutrient content to enhance population stability and expansion^60^. In the present study, we observed a close relationship between Ca^2+^ addition and egg diameter, with females producing more offspring in response to shell breakage to ensure population growth. However, our study solely investigate the calcium content of snails under one-time shell breakage stress with calcium carbonate addition and subsequent repair process in laboratory. Therefore, further research is warranted to elucidate the effects of the frequent shell breakage and repair process on snail population development, growth, and invasion in fields. In addition, exploring related mechanisms, such as gene expression, is imperative for a comprehensive understanding of this phenomenon.

## Conclusion

This study showed that when *P*. *canaliculata* facing with shell damage, the addition of calcium carbonate in water increased the shell repair rate of female snails in the earlier period, and then the rate gradually decreased. Food intake of *P. canaliculata* exhibited different patterns when the shell was broken and calcium was added, which is affected by the interaction between calcium carbonate addition and shell breakage. The addition of calcium resulted in a significant increase in calcium content in the mantle of females with shell breakage, which resulted in the increase of the shell strength, amounts and size of eggs, but no difference for males with shell breakage among the treatments. Therefore, snails are able to repair themselves rapidly through the biomineralization of Ca^2+^ in the water environment and food intake after suffered shell damage and are able to maintain the stability of their populations and the adoption of different survival strategies. This findings may provide some references for probing invasion mechanism of *P. canaliculata* and help to implement a more targeted prevention and control on them.

### Supplementary Information

Supplementary Information.

## Data Availability

All data generated or analysed during this study are included in this published article.
